# Shift-Work Schedule Intervention for Extending Restart Breaks after Consecutive Night Shifts: A Non-randomized Controlled Cross-Over Study

**DOI:** 10.3390/ijerph192215042

**Published:** 2022-11-15

**Authors:** Tomohide Kubo, Shun Matsumoto, Shuhei Izawa, Hiroki Ikeda, Yuki Nishimura, Sayaka Kawakami, Masako Tamaki, Sanae Masuda

**Affiliations:** 1 National Institute of Occupational Safety and Health, Nagao 6-21-1, Tama-Ku, Kawasaki 214-8585, Japan; 2 Research Center for Child Mental Development, Hamamatsu University School of Medicine, Handayama 1-20-1, Higashi-ku, Shizuoka 431-3192, Japan; 3 Cognitive Somnology RIKEN Hakubi Research Team, RIKEN Cluster for Pioneering Research, Hirosawa 2-1, Wako, Saitama 351-0198, Japan; 4 Cognitive Somnology RIKEN Hakubi Research Team, RIKEN Center for Brain Science, Hirosawa 2-1, Wako, Saitama 351-0198, Japan; 5 Nursing Department, Kanto Rosai Hospital, Kizukisumiyoshi 1-1, Nakahara-ku, Kawasaki 211-8510, Japan

**Keywords:** shift-work scheduling, occupational-fatigue counseling, participatory approach

## Abstract

More knowledge is required to determine the optimal shiftwork schedule to reduce the harmful effects of short restart breaks between shifts. This 5-month intervention study aimed to examine the effectiveness of extended restart breaks from 31 h to 55 h after consecutive night shifts by considering the characteristics of the circadian rhythm to mitigate fatigue and sleep among 30 shift-working nurses. Subjective and objective variables, such as vital exhaustion, distress, hair cortisol, salivary C-reactive protein, and sleep mattress sensor sensation, were repeatedly measured to examine the differences between the intervention and control conditions. Two-way (condition × time) multilevel analyses showed significantly lower levels of vital exhaustion and distress in the intervention condition (*p* = 0.005 and *p* = 0.004, respectively). However, the expected benefit of the intervention was not observed in objectively measured variables. These findings suggested that an extended restart break after consecutive night shifts can moderately decrease occupational fatigue and stress.

## 1. Introduction

In terms of the psychological and physiological aspects, shift workers could have a greater risk of diseases and accidents than daytime workers because they work at an inappropriate time in the circadian rhythm [1]. The harmful effects of shift work on employees are well known. For instance, much attention has been paid to suggesting a link between shift work and breast cancer [2] in addition to the conventional health consequences of shift work, such as fatigue and sleep [3]. In addition, a previous prospective cohort study reported that shift work chronically leads to impaired cognitive function, and recovery takes approximately five years after retiring from shift work [4]. In terms of occupational safety, an elevated risk of occupational injury was observed on workdays with evening shifts and workdays following night shifts [5]. However, shift work is inevitable for sustaining the social and economic aspects of society. To fill this gap, additional knowledge is required to determine the optimal shiftwork schedule to reduce its harmful effects. 

The speed and direction of shift rotation, distribution of days off, and employee worktime control within the shift system are relevant in relation to recovery and health [6]. Among these factors, sufficient sleep opportunities play a critical role in decreasing the health consequences of shift work [1]. This study focused on restart breaks, defined as the time for recuperation between duty cycles [7,8], as a strategic measure to ensure sleep opportunities. In addition, although there are some similar terms regarding restart breaks (e.g., quick returns (less than 11 h between shifts) and shift intervals), this study used the term “restart break”.

To improve shiftwork conditions, nine recommendations provided by Knauth and Rutenfranz have been agreed upon in this field [9]. Of these, avoiding a short restart break between two shifts has been addressed in recommendations since the 1980s. However, empirical data have recently accumulated to suggest an association between short restart breaks (i.e., less than an 11 h rest period between shifts) and negative health outcomes [10]. Moreover, the newly updated recommendations by Nordic researchers have suggested that night-shift schedules should be made with (i) ≤3 consecutive night shifts, (ii) restart breaks of ≥11 h, and (iii) a shift duration of ≤9 h to reduce the risk of occupational safety and health issues [11]. They also emphasized the importance of ensuring a sufficient restart break between the shifts.

Ensuring a sufficient restart break between shifts leads to more sleep opportunities for shift workers [7]. Lack of sleep can cause sleepiness and fatigue [12,13] in the short term, whereas its chronic effects can lead to several diseases [14] and mortality [15,16] in the long term. Furthermore, a previous study showed that for some variables (e.g., overworking and working night shifts), shorter sleep duration was the most effective variable for predicting the potential risks of overwork-related death in truck drivers [17]. According to a previous systematic review, shorter sleep duration, which is defined as a sleep duration of less than 5 h per night, could be linked to a greater risk of disease [18]. Collectively, these findings indicated that protecting sleep opportunities plays a critical role in health at work. 

Given that the physical and mental workloads could be greater during night shifts than during day shifts [19,20], a longer restart break should be arranged after night shifts than after day shifts. In laboratory research, a 34 h restart break was found to be sufficient in maintaining optimal alertness from one simulated duty cycle to the next when duty periods were scheduled during the day, but this was insufficient in preventing the build-up of fatigue across duty cycles when duty periods were scheduled during the night [7]. This plausibility was also supported by a two-week sleep diary study of 67 shift-working nurses [21]. The study showed that the sleep duration in a restart break of less than 11 h could be changed according to the combination of shift schedules (e.g., evening-to-day shifts or evening-to-evening shifts), decreasing (or increasing) with the circadian rhythm. A positive correlation between sleep duration and the length of restart breaks between shifts was also reported in a time-budget study [22]. Hence, the arrangement of restart breaks should be determined by considering circadian rhythm characteristics. 

Shift-work schedules can significantly affect circadian rhythms among shift workers [23,24]. In general, nurses’ shift-work schedules can be divided into at least two schedules: a three-shift schedule (day shift, evening shift, and night shift) and a two-shift schedule (day shift and night shift). Two three-shift schedules rotate in a forward direction (day shift to evening shift to night shift) and a backward direction (night shift to evening shift to day shift). Earlier studies have suggested that a backward rotation could be more difficult to adapt to than a forward rotation because the circadian rhythm tends to move forward [25,26]. For example, compared with forward-rotating schedules, backward-rotating shift systems showed a relative risk of 2.9 due to an increased need for recovery and a risk of 3.2 due to poor general health [27]. In Japan, more hospitals adopt a three-shift schedule over a two-shift schedule (56% vs. 44%, respectively) [28]. In addition, nurses working in three shifts with a backward rotation often complain about the arrangement of two consecutive night shifts (N) followed by two consecutive evening shifts (E) (i.e., NNEE), possibly due to the insufficient restart break to recover with after two consecutive night shifts. The union also reported that the arrangement of NNEE is often included in three 8 h shift schedules with a backward rotation. Thus, we hypothesized that allocating one additional off-duty day (O) after two consecutive night shifts (i.e., NNOEE), considering the circadian rhythm, would improve fatigue and stress at work. In addition, it is expected that this intervention could increase the shift interval between night and evening shifts and increase sleep opportunities without increasing the overall working hours.

On the other hand, in previous interventional studies, a major change in shift schedules required greater effort from the employer and employees, such as that in the direction of the shift rotation [29,30] and the shift length [31,32]. By contrast, changing the allocation of days off without increasing working days did not require major changes in this study. Given this background, this intervention may be more practical than previous ones. To the best of our knowledge, a controlled intervention study involving an extended restart break targeting shift-working nurses has not been conducted. Also, it is difficult to uniquely identify occupational fatigue using a single measure [33,34,35]. Therefore, this study subjectively and objectively measured fatigue-, stress-, and sleep-related variables, such as working hours, the sleep log, vital exhaustion and psychological distress, hair cortisol, salivary C-reactive protein, sleep mattress sensor sensation, and the psychomotor vigilance task, to examine the benefit of extended restart break. This 5-month intervention study using a crossover design aimed to determine if the effects of extending the restart break from 31 h to 55 h after two consecutive night shifts, considering the characteristics of the circadian rhythm, were beneficial for fatigue and sleep in shift-working nurses.

## 2. Materials and Methods

### 2.1. Participants

Based on the eligibility criteria, 37 nurses who worked as the head nurse in a cardiac surgery ward were recruited through a counter partner. These head nurses consulted our research team to improve working conditions among shift-working nurses. Thus, participants were recruited through these head nurses in this study. The criteria were (1) age ranging from 20 to 50 years, (2) having more than 3 years of work experience, (3) being an individual without current medical treatments, and (4) being a nurse currently working the three-shift schedule with backward rotation. Consequently, 30 shift-working nurses (age of 28.2 ± 5.9 years, work experience of 6.4 ± 5.6 years, body mass index of 20.4 ± 3.1, one-way commuting time of 20.7 ± 22.4 min; of the participants, 28 were single, and 2 had a child younger than 12 years old) participated in this study. However, one participant withdrew in January 2021 for personal reasons. Moreover, six participants contracted COVID-19 in January but recovered and returned to work without after-effects in February. The local institutional review board reviewed and approved the study protocol (2021N15). This study was registered in the UMIN Clinical Trials Registry (UMIN000041804). All participants provided written informed consent and received payment for their participation (120,000 JPY).

### 2.2. Study Design

**Figure 1:**
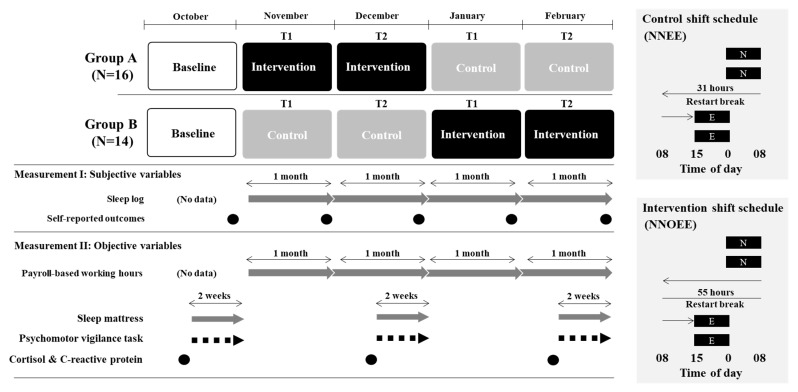
Study design. The evening shift (E) was from 15:45 to 0:30; the night shift (N) was from 0:00 to 8:45; and the off duty (O) was the first half period during intervention or control (T1) and the second half period during intervention or control (T2). Black circles represent the timing of measurement; gray arrows represent repeated measurement; and black dash arrows represent measurement after each shift.

As shown in Figure 1, the 5-month intervention study was conducted from October 2020 to February 2021 using a non-randomized crossover design, and the baseline period was set as October 2020. The participants were divided into two groups based on their original teams because they originally worked in two teams. In addition, their areas of responsibility differed between the two teams although they worked in the same ward. Therefore, it was difficult to allocate them randomly to groups A or B. Therefore, this study did not adopt a randomized controlled trial design. For the first period, from November to December 2020, groups A and B were allocated to the intervention and control conditions, respectively. Subsequently, these conditions were switched in the second period, from January to February 2021. T1 and T2 represent “the first half period during control or intervention” and “the second half period during control or intervention”.

### 2.3. Intervention

Before planning this intervention, occupational-fatigue counseling was conducted using a participatory approach which is defined as the process by which a practitioner (i.e., the researcher in this study) discusses with local employees to find locally workable solutions. In particular, occupational-fatigue counseling is regarded as an effective means to find a way to reduce work-induced fatigue levels. We interviewed four nurses who worked at the hospital to collect information about fatigue problems in relation to shift schedules. Counseling was conducted twice for about one hour at two times (December 2019 and February 2020). Nurses were then interviewed in terms of a problematic shift schedule by providing an actual shift roster. Consequently, anecdotal information, where many nurses complained about the shift schedule combination of NNEE, was obtained. We then discussed the optimal shift schedule with the head nurses in hospital wards to reduce fatigue among nurses. Based on this information, an intervention plan in which one off-duty day is allocated after two consecutive night shifts (i.e., NNOEE or NNOE) was formulated to ensure a restart-break period between shifts. The shift schedule was created by the head nurses in hospital wards based on the intervention plan.

### 2.4. Measurements

There is some evidence that occupational fatigue can be difficult to uniquely identify using conventional measures, as non-occupational sources can affect occupational-fatigue levels [33,34,35]. Furthermore, there are no measures that can holistically capture all the elements of occupational fatigue. Therefore, to multidimensionally assess occupational fatigue, this study conducted both subjective and objective measurements. With measurement I, the sleep log and self-reported questionnaire were measured using subjective variables. On the other hand, payroll-based working hours, sleep mattress sensor sensations, psychomotor vigilance tasks, and stress biomarkers were measured using objective variables with measurement II. In measurement II, sleep mattress sensors and psychomotor vigilance tasks were measured in the second half of October-December 2020 and February 2021, respectively.

#### 2.4.1. Measurement I: Subjective Variables

- Sleep log

Participants were required to record their sleep on a daily log in 30 min intervals for four months. Thus, there were 48 cells per day. Additionally, participants were required to report their sleep duration with each 30 min unit. To determine the effect of the intervention on sleep patterns, the monthly total sleep hours, defined as the summed value of sleep hours during each month, was calculated. The data were adjusted according to the number of days in each month. In addition, sleep data from restart breaks from night to evening shifts were analyzed in control and intervention conditions. The total sleep hours, the mean sleep duration, and the number of sleep opportunities were calculated during restart breaks. This study defined total sleep hours as the summed value of sleep hours across all night to evening shifts, mean sleep duration as the mean value of sleep duration across all night to evening shifts, and the number of sleep opportunities as the total number of nighttime sleeps across all night to evening shifts.

- Self-reported outcomes

The participants were instructed to answer the web questionnaire (Google Forms (paid version), Google LLC, CA) using their smartphones at the end of each month. The questionnaire asked about vital exhaustion (measured with the 21-item Maastricht Questionnaire [36]), psychological detachment from work (measured with the Recovery Experience Questionnaire [37]), psychological distress (measured using the 6-item Kessler Psychological Distress Scale [K6] [38]), and vigor (measured using the 9-item version of the Utrecht Work Engagement Scale [39]). Approximately 10 min were required to complete the questionnaire.

- Vital exhaustion

Vital exhaustion was defined as unusual fatigue, energy loss, increased irritability, and feelings of demoralization [40,41]. Vital exhaustion was assessed using the 21-item Maastricht Questionnaire, with questions such as “Do you often feel tired?” The responses were summed to obtain an overall vital exhaustion score ranging from 0 to 42, with higher scores indicating more exhaustion.

- Psychological detachment from work

Psychological detachment from work was defined as a state in which people mentally disconnect from work, and it was an important factor in their recovery from work [42]. The Recovery Experience Questionnaire consists of four subscales: psychological detachment (four items), relaxation (four items), control (four items), and mastery (four items). It was used to measure psychological detachment from work [37]. All items were scored on a 5-point Likert scale ranging from 1 (“do not agree at all”) to 5 (“fully agree”) that used statements such as “I do not think about work at al.” We summed and averaged the responses for each subscale to obtain the average score for each subscale.

- Psychological distress

The 6-item Kessler Psychological Distress Scale (K6) is a globally used questionnaire to measure psychological distress, and it includes six questions about emotional states [38]. All items are scored on a 5-point Likert scale ranging from 1 (“none of the time”) to 5 (“all of the time”) that used questions such as “During the past month, how often did you feel nervous?” Higher total scores indicate higher levels of psychological distress.

- Vigor

Work engagement was defined as a positive, fulfilling, work-related state of mind that is characterized by vigor, dedication, and absorption [43]. The 9-item version of the Utrecht Work Engagement Scale [39] was used. Vigor was assessed using three questions, such as “At work, I feel bursting with energy,” on a 7-point Likert scale ranging from 0 (“never”) to 6 (“always”).

- Shift schedule evaluation

To measure fatigue-related outcomes in relation to the shift schedule, we used a questionnaire developed for the current study. At the end of the intervention, the participants were required to evaluate the intervention shift schedule and control schedule on a 4-point Likert scale from 1 (“very low”) to 4 (“very high”) that used statements such as “Please rate your fatigue level from ‘very low’ to ‘very high’ for both the intervention shift and control schedules”. The evaluation items included fatigue, stress, sleepiness, quality of sleep, quality of care, satisfaction with days off, and work satisfaction.

#### 2.4.2. Measurement II: Objective Variables

- Objectively recorded working-hours data

This study included data on payroll-based working hours recorded by the hospital. In this study, daily working hours, monthly total working hours, number of days off, and number of quick returns (i.e., less than an 11 h restart break between shifts) were calculated. Monthly working hours were adjusted according to the number of days in each month. Daily working hours, including overtime and excluding legal break time (i.e., 1 h), were calculated. In addition, the restart-break length of night to evening shifts in the control and intervention conditions (i.e., control, NE, and intervention, NOE) was analyzed to determine the time change in the intervention shift schedule.

- Sleep mattress

Objectively measured sleep was measured using sleep mattress sensors (Nemuri SCAN system, Paramount Bed Co., Ltd., Tokyo, Japan) for each two-week period. Given that sleep logs have potential risks of overestimating (or underestimating) sleep, the data were reinforced by a sleep mattress. This non-wear actigraph had a highly sensitive pressure sensor and was placed under a mattress. It could continuously record the activities of a person lying on the mattress and identify an “in-bed/out-of-bed” state from the vibrations of the mattress. A validation study showed that the agreement rate of the scored sleep period was approximately 92.0 % between the device and polysomnography (PSG) [44]. The epoch length was set as 1 min. Total sleep time, sleep latency, sleep efficiency, and wake time after sleep onset were calculated.

- Psychomotor vigilance task

The psychomotor vigilance task (PVT) (PVT-192, Ambulatory Monitoring, Ardsley, NY, USA) was used to measure fatigue for each two-week period. The PVT is a simple reaction-time test in which participants are required to push a button as soon as a light appears. The participants were instructed to complete the PVT in the break room immediately after their work. We counted lapses (response latency exceeding 500 ms; data transformed by SQR [x] + SQR [x + 1]) and speed (1/mean reaction times × 1000) during each 5 min test, and we set a random interstimulus interval of 2 to 10 s.

- Stress level

Salivary C-reactive protein (CRP) and hair cortisol levels were measured three times (October 2020, December 2020, and February 2021) at the hospital to examine the level of objective stress. Saliva was collected using Salivette (Sarstedt, Ltd., Leicester, UK) polypropylene and polyethylene polymer swabs. The participants were required to place the swab under their tongues for at least 3 min to obtain the sample under the supervision of the researchers. This study adjusted CRP levels for total protein concentrations (CRP (pg) divided by total protein (mg)). Moreover, multiple strands of hair were collected from the posterior vertex as close as possible to the scalp using scissors to measure the hair cortisol levels. Because hair grows at approximately 1 cm per month, we collected 1 cm segments that were closest to the scalp. A specialist (SI) conducted the data analyses in our institute’s laboratory according to a previous study [45]. Detailed information is provided in the Supplementary Methods (Method S1).

### 2.5. Data Analyses

Data on self-reported outcomes, PVT, fatigue, sleep, and objectively measured stress were transformed to delta values from baseline measurements (i.e., T1-BL and T2-BL; data before transforming delta values are reported in Supplementary Tables (Supplementary Tables S1–S3)). The payroll data, sleep log, and shift schedule evaluation were analyzed using actual data because we did not measure these parameters at the baseline. Two-way (condition (control, intervention), time (T1, T2)) multilevel analyses were used to evaluate the effects of condition and time on self-reported outcomes and payroll data, and a one-way (condition (control, intervention)) multilevel analysis of objectively measured stress was conducted. In contrast, the data regarding PVT and sleep mattress sensors were analyzed using two-way (condition (control, intervention) and shift (day, evening, night, and days off)) multilevel analyses. This study included conditions and shifts as fixed factors and entered each participant as a random factor. Then, age, work experience, marital status (married/single), chronotype (obviously active in the morning = 1, somewhat active in the morning = 2, somewhat active in the evening = 3, and obviously active in the evening = 4), commuting time, month (Oct., Nov., Dec., Jan., and Feb.), group (A, B), care for COVID-19 patients during the study period (yes/no), COVID-19 anxiety (very low = 1, very high = 5), and COVID-19 infection (yes/no) were adjusted as covariates. In addition, wake time, measurement time (before or after shift), and K6 were also adjusted as covariates in the analysis of the salivary CRP. Moreover, use of hair dye (yes/no) was added as a covariate when analyzing hair cortisol. As mentioned above, one participant retired for personal reasons in January 2021, and six participants contracted COVID-19 in January 2021 (but returned to work without after-effects in February). Therefore, this study treated these data as missing values and adopted a multilevel analysis because this statistical method did not require missing values to be filled in. However, this study treated these data as lost data when there were missing data in both the baseline and other measurements (i.e., T1 and T2). Meanwhile, we analyzed the data on payroll-based shift interval length, sleep log, and subjective shift evaluation using a paired t-test. Statistical analyses were performed using IBM SPSS Statistics version 26 for Windows. Statistical significance was set at *p* < 0.05.

## 3. Results

### 3.1. Characteristics of the Intervention Shift Schedule

Table 1 shows that the intervention shift schedule affected working hours, log-recorded sleep, and subjective shift evaluation. Regarding the shift combinations recorded in the payroll data, NNEE in the control condition (mean (SEM); T1 = 1.09 [0.05], T2 = 1.24 [0.08]), NNOE (T1 = 1.35 [0.09], T2 = 1.19 [0.07]), and NNOEE (T1 = 1.07 [0.05], T2 = 1.00 [0.00]) in the intervention condition were approximately once per month. In addition, the number of each shift and days off per month was as follows in the intervention condition: for day shift T1 was 8.03 [0.56] and T2 was 9.90 [0.34], for evening shift T1 was 3.41 [0.23] and T2 was 3.83 [0.23], for night shift T1 was 3.34 [0.22] and T2 was 3.17 [0.20], and for days off T1 was 13.0 [1.17] and T2 was 12.4 [0.80]. In the control condition, they were as follows: for day shift T1 was 9.63 [0.44] and T2 was 9.77 [0.28], for evening shift T1 was 4.00 [0.21] and T2 was 3.77 [0.21], for night shift T1 was 3.23 [0.20] and T2 was 3.17 [0.20], and for days off T1 was 13.4 [1.22] and T2 was 12.4 [0.60]. Multilevel analyses showed significant effects of time (T1 vs. T2) on working hours (F1, 76.225 = 13.877, *p* < 0.001), monthly total working hours (F1, 81.033 = 5.265, *p* = 0.024), days off (F1, 80.478 = 11.236, *p* = 0.001), and monthly total sleep hours (F1, 76.230 = 6.648, *p* = 0.012). Therefore, significantly longer working hours, fewer days off, and shorter sleep hours were observed in T2 over T1. However, we found no main effect of the condition or interaction between the condition and time. Meanwhile, as shown in Table 2, the intervened night to evening shift had a significantly longer shift interval length in the intervention condition than in the control condition (t26 = −156.179, *p* < 0.001). Regarding the sleep log, significantly longer total sleep hours and more sleep opportunities were observed in the intervention condition than in the control condition (t24 = −14.261, *p* < 0.001, and t24 = −8.406, *p* < 0.001, respectively), but no significant difference between the control and intervention conditions was found in mean sleep duration. Taken together, it was confirmed that this intervention schedule led to increased shift intervals and sleep opportunities without increasing overall working hours.

### 3.2. Intervention Effects on Self-Reported Outcomes

**Figure 2:**
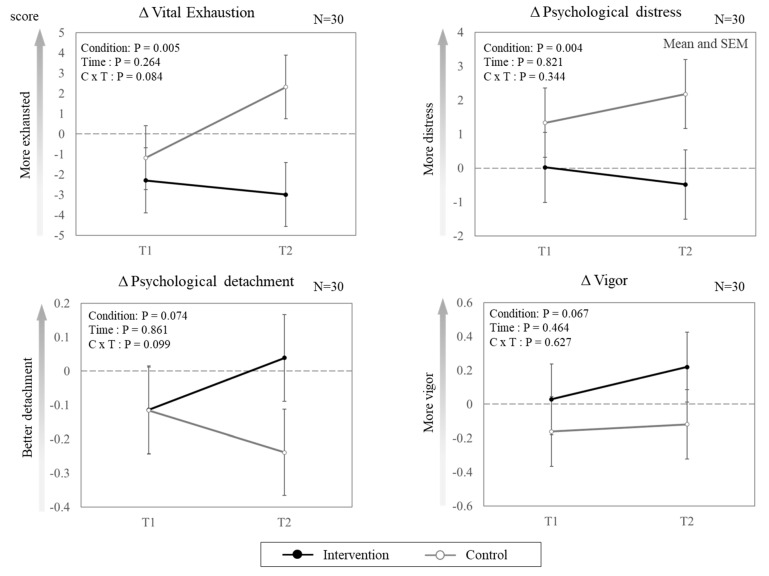
Effect of intervention shift schedule on self-reported outcomes (marginal means and SEM). The data represent the delta values from baseline measurements (i.e., T1-BL and T2-BL). The dotted line represents the baseline.

Figure 2 presents the effects of the intervention shift schedule on the self-reported outcomes. Of the four outcomes, vital exhaustion and psychological distress showed significant effects on condition (F_1, 79.754_ = 8.239, *p* = 0.005; F_1, 80.623_ = 8.853, *p* = 0.004, respectively). However, significant differences between the two conditions were not observed for psychological detachment or vigor. Regarding the effect of time, no significant differences were found among the four outcomes. Significant interactions between the condition and time were not observed. Regarding subjective shift evaluation, significantly better outcomes for fatigue, stress, sleepiness, quality of sleep, satisfaction with days off, and work satisfaction were found in the intervention condition than in the control condition (ps < 0.001) (Table 3). However, no significant difference in the quality of care was found between the two conditions.

### 3.3. The Intervention’s Effects on Salivary CRP and Hair Cortisol

**Figure 3:**
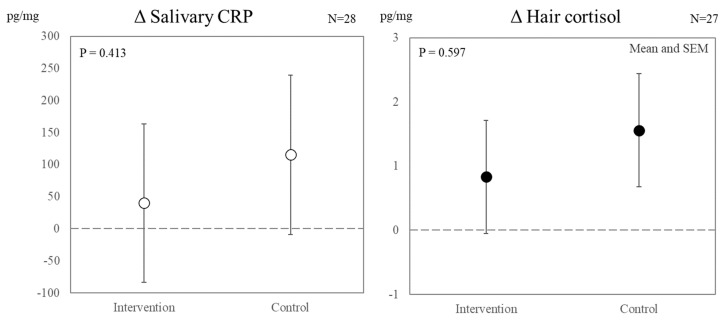
Effect of intervention shift schedule on objectively measured stress (marginal means and SEM). The data represent the delta values from baseline measurements (i.e., T1-BL and T2-BL). The dotted line represents the baseline.

Figure 3 presents the effects of the intervention shift schedule on objectively measured stress. A one-way multilevel analysis showed no significant differences in salivary CRP or hair cortisol levels between the two conditions. 

### 3.4. The Intervention’s Effects on Objectively Measured Fatigue and Sleep during the Two-Week Observation

Table 4 presents the effects of the intervention shift schedule on fatigue and sleep outcomes during the two-week observation. Regarding the PVT and sleep mattress sensors, no significant main effects (of condition and time) were observed. 

## 4. Discussion

This study examined the benefits of extending the restart break in fatigue and stress after two consecutive night shifts, considering the circadian rhythm, among shift-working nurses. In the intervention shift schedule, the allocation of days off was changed without changing working hours or quick returns, thereby increasing sleep opportunities during the restart break (Table 1 and Table 2). Consequently, nurses who experienced the intervention shift schedule showed significantly less exhaustion and distress than their counterparts. However, no obvious benefit of the intervention was found in objectively measured stress, sleep, or PVT performance (see Figure 3 and Table 4).

As expected, this study observed the favorable effects of the intervention shift schedule with an extended restart break on some outcomes, especially self-reported exhaustion, distress, and shift schedule evaluation. These findings are partially consistent with those of a previous study that examined the benefits of extending restart breaks from 7 to 11 h between shifts for vehicle drivers [46]. An earlier study also suggested that the positive effect of extended restart breaks was more obvious in self-reported outcomes than objective outcomes. As discussed in the study, the authors pointed out that these modest benefits stemmed from banked sleep during extended restart breaks [47]. Since our data did not show an increase in mean sleep duration, our findings could not be supported by the sleep banking hypothesis. However, the definition of sleep banking is lacking according to a systematic review [48]. It has been discussed whether sleep banking should refer only to the major sleep period or to all sleep obtained in a 24 h period, including napping. If the latter definition is broadly accepted, the increased sleep opportunities and total sleep hours observed in this study may be regarded as a variant of sleep banking. Hence, given that one additional night of sleep was ensured by extending restart breaks in this study (Table 2), the cumulative effects of two consecutive night shifts could be mitigated by sleep banking before working a subsequent evening shift. 

Moreover, as mentioned above, this study set the interventional target based on the fact that nurses most frequently complained about shift combination (i.e., NNEE) in the occupational-fatigue counseling conducted before launching this study in order to examine the content of the intervention with local employees. Consequently, nurses who work on an interventional shift schedule could easily realize these benefits. Additionally, significantly higher levels of shift schedule evaluation with the intervention shift schedule could stem from the participatory approach involving occupational-fatigue counseling (Table 3). Meanwhile, a previous intervention study regarding shift schedule changes among air traffic controllers indicated that the employee’s disagreement with the schedule changes could be one of the causes for the experiment not resulting in the expected beneficial effects [49]. Taken together, these findings suggest that the methodology of occupational-fatigue counseling based on the participatory approach could be a key factor in determining the content of the intervention.

Notably, this intervention did not lead to increased objectively recorded working hours, including overtime hours. If increased working hours or decreased sleep hours occurred due to the intervention, it was regarded as a negative influence. However, this intervention had a favorable effect on some outcomes without those negative influences. In contrast, previous interventional studies have greatly changed the shift schedule, such as the direction of the rotation from backward to forward rotations [30], the speed of rotation from a backward to a very quick forward shift rotation [29], and the shift length from an 8 h to a 12 h shift system [31]. These major changes have led to positive outcomes. However, greater effort from employers and employees could be required. Therefore, considering our results, without major changes in the shift schedule, the feasibility of introducing the intervention shift schedule in other hospitals with a schedule involving three 8 h shifts with a backward rotation could be relatively higher than that in previous studies. 

In addition, accumulated data suggest that shift workers can better adapt to forward rotations than backward rotations in terms of the circadian rhythm [27,50,51]. In particular, the health consequences of circadian disruption, such as breast cancer [52] and type 2 diabetes [53], could be pointed out by previous research. However, this intervention shift schedule was conducted by changing the allocation of the days off while maintaining a backward rotation. More beneficial effects would be expected if the intervention was completely changed from backward to forward rotations. On the other hand, the positive outcomes observed in this study could likely be attributed to a specific shift combination (i.e., NNEE) that results in circadian disruption. In terms of circadian adaptation to shift work, comparing a conventional shift schedule intervention toward a forward rotation with this study in a future investigation with long observation periods may provide new insights into understanding how shift schedule interventions should be planned. Namely, the findings would be very practical if better outcomes were obtained by a tailor-made intervention with occupational-fatigue counseling over a conventional shift schedule intervention with major changes. 

The important question here is why this study did not obtain significant findings in the objectively measured outcomes. A systematic review of shift-work interventions [54] suggested that out of 15 interventional studies on shift schedule changes, 3 showed significant positive changes in objective outcomes, such as actigraphic sleep [29], triglycerides [51], and resting blood pressure [30] compared to the control group. Namely, those previous studies showing the benefits of objective outcomes were conducted with longer observation periods (i.e., between 1 and 2 years) and larger samples (i.e., between 84 and 140 participants). Thus, it may be reasonable to conclude that this study did not find significant differences in the objective outcomes. Therefore, further studies solving those possible issues are necessary to examine the effects of an intervention shift schedule with an extended restart break on objective outcomes.

### Strengths and Limitations

One strength of this study was that the participatory approach, including occupational-fatigue counseling, was effective because researchers could determine the content of the intervention based on the estimation of risk resulting from shift work by local employees. Moreover, in this intervention shift schedule, only the allocation of days off was changed, without increasing overtime during the study period. Notably, some wards in the participating hospital decided to implement an intervention shift schedule after this study. This data-based fact boosts the feasibility of implementing this shift schedule in other hospitals with a schedule involving three 8 h shifts with a backward rotation.

However, this study had some limitations. First, compared to previous studies on shift schedule intervention, this study included a smaller sample and shorter observation period [54], which may be the reason this study did not have positive effects on objective outcomes. In addition, it is possible that this intervention, involving the introduction of a few extra days off per month, could not change the level of objective outcomes (esp. hair cortisol and salivary C-reactive protein) to a noticeable degree regardless of some possible confounding factors. Also, the question of why this study could not detect the effect of this intervention on objectively measured sleep may be related to the validity of the sleep mattress sensors since the only study comparing these sensors and polysomnography is based on six subjects and also showed quite a low specificity. Second, the generalizability of the findings could be limited because this study was conducted in a single hospital ward. However, our findings could be relevant in a hospital with three 8 h shifts with a backward rotation. Third, the participants may have suffered from COVID-19-related stress. Six participants contracted COVID-19 in January 2021. At that time, vaccination had not started in Japan. Moreover, the risk of COVID-19 infection remains unknown. Given these situations, participating nurses could have suffered from severe stress compared to the normal working situation. COVID-19-related stress could likely have affected our data. Further research should be conducted in a normal working situation after the COVID-19 pandemic to assess these findings’ validity. Fourth, this study could not establish a washout period between the intervention and control conditions. This may have affected the results for those going from intervention to control. Fifth, as mentioned above, a backward rotation is not an appropriate shift system in terms of the circadian rhythm [25,26]. The shift schedule intervention basically remained as a backward rotation while this intervention study extended restart breaks after consecutive night shifts. Although some possibly confounding factors were adjusted in the analyses, they could be related to the reason why this study could not observe the benefits of this intervention on objective outcomes. Sixth, we evaluated fatigue-related outcomes in relation to the shift schedule with the questionnaire developed for this study. However, a valid and widely used scale (e.g., Karolinska Sleepiness Scale [55] and Tiredness Symptoms Scale [56]) should be used to compare our findings with other studies. Of the possible appropriate questionnaires, the Standard Shiftwork Index [57] was thought to be a good tool, but there was no validated Japanese version. Further investigations using such appropriate questionnaires are needed to test the current findings. Finally, we must address the positive and negative aspects of occupational-fatigue counseling. The positive aspects have already been addressed above. On the other hand, participants could have overestimated the effectiveness of the intervention because the workplace-fatigue counseling involved a procedure to reflect opinions from local employees. However, the procedure would inevitably lead to actual changes in the working conditions on site, even after this intervention.

## 5. Conclusions

Our findings suggested that the positive influences of a shift schedule with an additional off-duty day between two consecutive night shifts and evening shifts influenced fatigue and sleep among shift-working nurses. Beneficial effects were found in the subjective variables, especially for vital exhaustion, psychological distress, and shift schedule evaluation. However, the expected benefit of the intervention was not observed in the objectively measured variables. This intervention shift schedule could be achieved by only changing the allocation of days off without increasing overtime or the number of days off during the study period. Therefore, in terms of the required efforts from employers and employees, it is highly feasible to implement the intervention shift schedule in other hospitals whose schedules include three 8 h shifts with a backward rotation.

## Figures and Tables

**Table 1:** Overall characteristics of intervention effect on working hours and sleep.

		Control		Intervention		Multilevel Analysis					
		Marginal Mean	SEM	Marginal Mean	SEM	Condition		Time		Interaction	
						F	*p*	F	*p*	F	*p*
Payroll data (N = 30)											
Working hours	T1	9.7	0.2	9.9	0.2	1.297	0.258	14.736	<0.001	0.747	0.390
(including overtime)	T2	10.1	0.2	10.2	0.2						
Monthly total working hours	T1	163.5	4.7	161.8	4.7	<0.001	0.997	13.289	<0.001	0.252	0.617
(including overtime)	T2	174.2	4.7	175.9	4.6						
Quick returns (times;	T1	0.5	0.3	0.2	0.3	0.775	0.381	2.008	0.160	0.531	0.468
<11 h shift interval)	T2	0.6	0.3	0.6	0.2						
Sleep log (N = 30)											
Monthly total sleep hours (hours)	T1	204.7	9.3	206.3	9.2	0.731	0.395	8.808	0.004	1.604	0.209
	T2	197.8	9.2	189.1	9.1						

Note: Values in bold indicate significant differences. Monthly total sleep hours was defined as the summed value of sleep hours during each month.

**Table 2:** Specific characteristics of intervention effect on shift interval length and sleep.

From N to E	NE (Control)		NOE (Intervention)		*t* Test	
	Mean	SEM	Mean	SEM	*t*	*p*
Payroll data (N = 27)						
Shift interval length (hours)	29.1	0.7	53.1	0.9	−156.179	<0.001
Sleep log (N = 25)						
Total sleep hours (hours) a	13.7	2.2	21.8	2.9	−14.261	<0.001
Mean Sleep duration (hours) b	7.0	1.9	7.4	2.2	−1.079	0.291
Number of sleep opportunities (times) c	2.1	0.5	3.2	0.7	−8.406	<0.001

Note: Values in bold indicate significant differences. E = evening shift, N = night shift, and O = off duty. (a) Total sleep hours was defined as the summed value of sleep hours across all night to evening shifts. (b) Mean sleep duration was defined as the average sleep duration across all night to evenings shifts. (c) Number of sleep opportunities was defined as the total number of nighttime sleeps across all night to evening shifts.

**Table 3:** Comparison of shift schedule evaluations between control and intervention shift schedules.

Shift Schedule Evaluation (N = 28)	Control		Intervention		*t* Test	
(1 = low, 4 = high)	Mean	SEM	Mean	SEM	*t*	*p*
Fatigue	3.4	0.6	2.2	0.4	10.2	**<0.001**
Stress	3.2	0.6	2.2	0.7	5.9	**<0.001**
Sleepiness	3.2	0.8	2.2	0.7	4.9	**<0.001**
Quality of sleep	2.0	0.7	2.9	0.6	−6.4	**<0.001**
Quality of care	2.3	0.5	2.3	0.7	0.0	1.000
Day-off satisfaction	2.0	0.7	3.4	0.6	−7.9	**<0.001**
Work satisfaction	2.3	0.5	2.8	0.5	−4.5	**<0.001**

Note: Values in bold indicate significant differences.

**Table 4:** Effect of intervention shift schedule on psychomotor vigilance task, fatigue, and sleep mattress sensor sensation during the two-week observation.

	Shift Type	Control		Intervention		Condition		Shift Type		Interaction	
		Marginal Mean	SEM	Marginal Mean	SEM	F	*p*	F	*p*	F	*p*
PVT (N = 29)											
Δ Mean RRT	Day shift	−0.33	0.09	−0.38	0.09	3.284	0.073	1.113	0.332	1.670	0.193
	Evening shift	−0.26	0.09	−0.29	0.09						
	Night shift	−0.24	0.10	−0.52	0.10						
Δ Lapse	Day shift	0.84	0.32	0.83	0.33	1.415	0.237	2.254	0.110	1.207	0.303
(SQRT)	Evening shift	0.89	0.33	1.00	0.36						
	Night shift	1.01	0.37	1.82	0.37						
Sleep mattress (N = 29)											
Δ TST (hr)	Day shift	−0.05	0.51	0.12	0.51	0.109	0.742	1.095	0.352	.221	0.882
	Evening shift	−0.27	0.57	−0.55	0.55						
	Night shift	−0.83	0.56	−0.28	0.60						
	Day off	0.18	0.50	0.25	0.52						
Δ SL (min)	Day shift	0.66	2.66	5.13	2.65	0.239	0.625	1.908	0.131	.612	0.608
	Evening shift	1.87	2.95	1.52	2.84						
	Night shift	−0.55	2.90	0.45	3.08						
	Day off	−1.62	2.62	−2.99	2.69						
Δ SE (%)	Day shift	−1.24	1.30	−1.36	1.30	0.690	0.408	1.072	0.363	0.163	0.921
	Evening shift	−2.92	1.46	−1.64	1.40						
	Night shift	−1.48	1.43	−0.03	1.53						
	Day off	−0.41	1.29	0.20	1.32						
ΔWASO (min)	Day shift	−2.04	7.80	0.50	7.80	0.510	0.476	1.270	0.287	0.810	0.490
	Evening shift	8.42	8.61	0.64	8.29						
	Night shift	3.65	8.48	18.20	8.96						
	Day off	−4.27	7.72	1.92	7.91						

Note: PVT = psychomotor vigilance task, RRT = reciprocal response time, SQRT = square root, TST = total sleep time, SL = sleep latency, SE = sleep efficiency, and WASO = wake after sleep onset.

## Data Availability

The data are not available because we did not inform the participants on data transparency, and we declare the possibility on the institutional review board.

## Supplementary Materials

The following supporting information can be downloaded at https://www.mdpi.com/article/10.3390/ijerph192215042/s1.
