# Supplementary material for: Shift-Work Schedule Intervention for Extending Restart Breaks after Consecutive Night Shifts: A Non-randomized Controlled Cross-Over Study
Source: Int J Environ Res Public Health. 2022 Nov 15;19(22):15042. doi: 10.3390/ijerph192215042 (PMC9691089; doi:10.3390/ijerph192215042)
Supplement: Supplementary file 1 [file ijerph-19-15042-s001.zip › Supplementary Method.pdf]

Supplementary Method

Objectively measured stress level

We measured salivary C-reactive protein (CRP) and hair cortisol three times (October and December of 2020, February of 2021) at the hospital to examine the level of objective stress. We collected saliva with a Salivette (Sarstedt, Ltd. UK) polypropylene and polyethylene polymer swab. The participants were required to place the swab under their tongue for at least 3 minutes to obtain the sample under the researchers' supervision. We also instructed them to refrain from eating, drinking, or brushing their teeth for 30 minutes before the collection. We stored the samples in a deep freezer as soon after collection as possible. We determined the concentration of CRP in the saliva using an enzyme immunoassay and an ELISA Kit (Salivary C-reactive protein ELISA kit, Salimetrics LLC, USA). The interassay and intra-assay variations were below 7% and 4%, respectively. In addition, we measured salivary total protein levels (mg/ml) using a Bradford Protein Assay Kit (Bio-Rad Laboratories, Inc., USA). We adjusted CRP levels for total protein concentrations (i.e., CRP [pg] divided by total protein [mg]).

We took multiple strands of hair from the posterior vertex as close as possible to the scalp using scissors to measure hair cortisol. Because hair grows approximately 1 cm per month, we collected the 1-cm segment closest to the scalp. We collected approximately 30 mg of hair segments for each measurement and stored the sample at room temperature and covered them with aluminum foil until the day we determined the cortisol level. The specialist (SI) conducted the data analyses in our institute's laboratory according to the previous study (Sugaya et al. 2020). We determined cortisol levels using an enzyme-linked immunosorbent assay (Cortisol EIA kit, Salimetrics LLC., USA). The interassay and intra-assay variations were less than 8.6 % and 4.4 %, respectively.

Sugaya, N.; Izawa, S.; Ogawa, N.; Shirotsuki, K.; Nomura, S., Association between hair cortisol and diurnal basal cortisol levels: A 30-day validation study. *Psychoneuroendocrinology* **2020**, *116*, 104650.
