# Supplementary material for: Shift-Work Schedule Intervention for Extending Restart Breaks after Consecutive Night Shifts: A Non-randomized Controlled Cross-Over Study
Source: Int J Environ Res Public Health. 2022 Nov 15;19(22):15042. doi: 10.3390/ijerph192215042 (PMC9691089; doi:10.3390/ijerph192215042)
Supplement: Supplementary file 1 [file ijerph-19-15042-s001.zip › Supplementary Tables.pdf]

Supplementary Tables

Table S1

Data about self-reported outcomes before transforming delta values from the baseline.

		**Baseline**		**Control**		**Intervention**	
**N=30**		**Mean**	**SEM**	**Mean**	**SEM**	**Mean**	**SEM**
Vital Exhaustion	BL	24.63	1.77	-	-	-	-
	T1	-	-	24.34	1.81	23.21	2.01
	T2	-	-	25.41	1.80	20.61	1.71
Psychological distress	BL	10.10	1.08	-	-	-	-
	T1	-	-	11.79	1.05	10.48	1.12
	T2	-	-	12.10	1.09	9.36	1.08
Psychological detachment	BL	3.27	0.17	-	-	-	-
	T1	-	-	3.08	0.18	3.15	0.15
	T2	-	-	3.05	0.17	3.33	0.16
Vigor	BL	2.48	0.24	-	-	-	-
	T1	-	-	2.33	0.24	2.46	0.22
	T2	-	-	2.37	0.23	2.79	0.21

Table S2

Data about objectively measured stress before transforming delta values from the baseline.

		Baseline		Control		Intervention	
	N	Mean	SEM	Mean	SEM	Mean	SEM
Salivary CRP (pg/mg)	28	267.88	43.48	298.42	67.36	249.92	42.12
Hair cortisol (pg/mg)	27	9.92	0.46	9.77	0.46	12.28	1.02

Table S3

Data about psychomotor vigilance task, fatigue, and sleep mattress before transforming delta values from the baseline

			Baseline		Control		Intervention	
		Shift type	Mean	SEM	Mean	SEM	Mean	SEM
PVT N=29	Mean RRT	Day shift	3.77	0.07	3.44	0.08	3.36	0.09
		Evening shift	3.84	0.09	3.61	0.09	3.51	0.10
		Night shift	3.80	0.07	3.47	0.08	3.24	0.09
	Lapse (SQRT)	Day shift	2.47	0.19	3.33	0.30	3.50	0.31
		Evening shift	2.10	0.20	2.92	0.31	3.25	0.35
		Night shift	2.14	0.21	3.21	0.29	4.17	0.33
Sleep mattress N=29	Total sleep time (hr.)	Day shift	7.33	0.31	7.18	0.36	7.46	0.38
		Evening shift	7.19	0.29	7.07	0.31	6.94	0.30
		Night shift	6.53	0.46	6.04	0.41	6.61	0.65
		Day-off	6.27	0.43	6.38	0.39	6.52	0.24
	Sleep latency (min)	Day shift	17.89	2.08	19.29	1.95	23.38	3.51
		Evening shift	12.78	0.81	16.44	2.06	14.82	1.58
		Night shift	14.58	1.12	13.63	1.14	16.15	1.79
		Day-off	20.93	3.10	19.90	2.13	18.09	1.84
	Sleep efficiency (%)	Day shift	89.98	0.72	88.56	0.95	88.29	0.83
		Evening shift	91.43	0.72	88.30	0.87	89.45	0.92
		Night shift	87.98	0.89	88.49	1.07	87.98	1.02
		Day-off	85.35	1.23	84.67	1.06	84.98	1.10
	WASO (min)	Day shift	31.24	5.17	29.19	3.52	33.75	4.75
		Evening shift	28.45	5.50	37.15	6.19	31.33	4.67
		Night shift	24.42	2.92	23.39	3.58	45.90	9.89
		Day-off	41.91	10.39	38.25	4.91	46.46	6.48

Note: PVT = psychomotor vigilance task, RRT = reciprocal response time, SQRT = square root, TST = total sleep time, SL = sleep latency, SE = sleep efficiency, WASO = wake after sleep onset. Values in bold indicate significant differences.
